# Estimated Burden of Coccidioidomycosis

**DOI:** 10.1001/jamanetworkopen.2025.13572

**Published:** 2025-06-03

**Authors:** Samantha L. Williams, Kaitlin Benedict, Brendan R. Jackson, Malavika Rajeev, Gail Cooksey, Irene Ruberto, Thomas Williamson, Rebecca H. Sunenshine, BreAnne Osborn, Hanna N. Oltean, Rebecca R. Reik, Michael S. Freedman, Andrej Spec, Adrienne Carey, Ilan S. Schwartz, Luis Medina-Garcia, Nathan C. Bahr, Rasha Kuran, Arash Heidari, George R. Thompson, Royce Johnson, John N. Galgiani, Tom Chiller, Mitsuru Toda

**Affiliations:** 1 Mycotic Diseases Branch, Division of Foodborne, Waterborne, and Environmental Diseases, National Center for Emerging and Zoonotic Infectious Diseases, Centers for Disease Control and Prevention, Atlanta, Georgia; 2 National Center for Immunization and Respiratory Diseases, Centers for Disease Control and Prevention, Atlanta, Georgia; 3 California Department of Public Health, Sacramento; 4 Arizona Department of Health Services, Phoenix; 5 Maricopa County Department of Public Health, Phoenix, Arizona; 6 Career Epidemiology Field Officer Program, Division of State and Local Readiness, Centers for Disease Control and Prevention, Atlanta, Georgia; 7 Utah Department of Health and Human Services, Salt Lake City; 8 Washington State Department of Health, Shoreline; 9 Michigan Department of Health and Human Services, Lansing; 10 Department of Pediatrics, Stanford University School of Medicine, Palo Alto, California; 11 Division of Infectious Disease, Washington University in St Louis School of Medicine, St Louis, Missouri; 12 Division of Infectious Diseases, Department of Internal Medicine, University of Utah School of Medicine, Salt Lake City; 13 Division of Infectious Diseases, Department of Medicine, Duke University School of Medicine, Durham, North Carolina; 14 University Medical Center of Southern Nevada, Las Vegas; 15 Division of Infectious Diseases and International Medicine, Department of Medicine, University of Minnesota, Minneapolis; 16 Division of Infectious Diseases, Department of Medicine, University of Kansas Medical Center, Kansas City; 17 Division of Infectious Diseases, Department of Medicine, Kern Medical Center–UCLA (University of California, Los Angeles), Bakersfield; 18 David Geffen School of Medicine, UCLA, Los Angeles; 19 Morehouse School of Medicine, Atlanta, Georgia; 20 Bakersfield Memorial Hospital, Dignity Health, Bakersfield, California; 21 Division of Infectious Diseases, Department of Internal Medicine, University of California Davis Medical Center, Sacramento; 22 Valley Fever Center for Excellence, Departments of Medicine and Immunobiology, College of Medicine–Tucson, BIO5 Institute, University of Arizona, Tucson

## Abstract

**Importance:**

Coccidioidomycosis is an underrecognized fungal infection that can cause serious illness and constitutes a considerable public health burden. The number of cases is likely substantially higher than the nationally reported total, as surveillance does not capture patients who do not seek medical care or who are undiagnosed or misdiagnosed. Coccidioidomycosis is not reportable in all states, and cases not reported to public health entities are likewise missed. A systematic estimate of coccidioidomycosis burden is needed to raise awareness and inform public health interventions and policy.

**Objective:**

To assess the annual burden of symptomatic coccidioidomycosis in the US.

**Design, Setting, and Participants:**

This cross-sectional study developed models incorporating coccidioidomycosis cases reported to the National Notifiable Diseases Surveillance System from January 1 to December 31, 2019, as model inputs. Multipliers from US public health surveillance accounted for factors including health care–seeking behavior, underdiagnosis, underreporting, and in-hospital mortality. Multiplier values were sourced from a combination of literature review and expert opinion. Regional estimates were generated using endemicity levels categorized as high (Arizona and California), low (Nevada, New Mexico, Texas, Utah, and Washington), or unknown (all other states and Washington, DC). Data were accrued from January 1, 2022, to July 1, 2024, and analyzed from October 1, 2022, to September 1, 2024.

**Exposure:**

Coccidioidomycosis reported to public health surveillance entities.

**Main Outcomes and Measures:**

Models estimated annual incident symptomatic coccidioidomycosis cases, hospitalizations, and deaths nationally and regionally in the US.

**Results:**

A nationwide total of 273 000 (95% credible interval [CrI], 206 000-360 000) incident symptomatic coccidioidomycosis cases were estimated in 2019. High-endemic states accounted for the highest burden (125 000 [95% CrI, 94 000-165 000] cases), followed by states of unknown endemicity (103 000 [95% CrI, 66 000-155 000] cases) and low-endemic states (46 000 [95% CrI, 31 000-65 000] cases). Nationally, models estimated 23 000 annual hospitalizations (95% CrI, 18 000-28 000) and 900 annual deaths (95% CrI, 700-1100) associated with coccidioidomycosis.

**Conclusions and Relevance:**

In this cross-sectional study, the estimated national burden of symptomatic coccidioidomycosis in 2019 was 10 to 18 times higher than the number of cases reported through national surveillance. Better awareness, diagnostic testing practices, and reporting are needed to improve patient outcomes and enhance our understanding of coccidioidomycosis epidemiology.

## Key Points

**Question:**

What is the annual burden of incident symptomatic coccidioidomycosis cases in the US?

**Findings:**

In this cross-sectional study using 2019 reported case data, models estimated the occurrence of 206 000 to 360 000 incident symptomatic cases of coccidioidomycosis in 2019 in the US, 10 to 18 times the number of cases reported through national surveillance. This study estimated 18 000 to 28 000 coccidioidomycosis-associated hospitalizations and 700 to 1100 coccidioidomycosis-associated deaths in 2019 nationwide.

**Meaning:**

These findings suggest that coccidioidomycosis burden estimates substantially exceed nationally reported case counts, highlighting the need for improved awareness, education, diagnostic testing practices, and reporting to inform public health efforts and achieve better patient outcomes.

## Teaser

This cross-sectional study generates a systematic and rigorous estimate of national incident symptomatic coccidioidomycosis cases by developing a model to account for cases missed through national surveillance.

## Introduction

Coccidioidomycosis, or Valley fever, is a fungal infection caused by the inhalation of *Coccidioides* species spores from soils of arid and semiarid regions in the western US, particularly Arizona and California.^1^ Although coccidioidomycosis is often asymptomatic or presents with self-limiting symptoms, nearly three-quarters of persons with coccidioidomycosis experience symptoms (eg, fatigue, pain, weakness) that limit daily activities.^2,3^ A small proportion (5%-10%) of people experience chronic illness, and approximately 1% develop disseminated disease. The annual economic burden resulting from direct medical costs and productivity losses is estimated to exceed $385 million in the US.^4^

Each year, approximately 10 000 to 20 000 cases are reported through US national surveillance, doubling from 8232 cases in 2014 to 20 003 cases in 2019.^5^ However, coccidioidomycosis is only reportable in 28 states and Washington, DC, and these totals likely represent only a fraction of the true national burden of coccidioidomycosis.^6,7,8^

Detection of coccidioidomycosis is complicated by several factors, including barriers to health care access, people who do not seek care, underdiagnosis, underreporting, and residence outside the known endemic region.^9^ People who experience mild symptoms may never seek care, making it impossible to identify them. For those who do seek care, the diagnosis is often delayed or missed.^2,3,10^ Coccidioidomycosis symptoms are nonspecific and may be indistinguishable from bacterial or viral community-acquired pneumonia (CAP).^11^ Clinician knowledge and awareness are often limited, and testing may be infrequent, especially outside the known endemic areas.^12,13,14^ The landscape of diagnostic testing can be difficult to navigate due to numerous test types (eg, antibody, antigen, culture, histopathology), logistical challenges (eg, biosafety precautions, lack of standardized serologic methods), and nuanced interpretations because test performance may vary based on factors related to the host, disease course, or specimen quality.^15,16^ Even when a patient is diagnosed accurately, cases are not always reported to public health authorities or may be misdiagnosed as prior disease and not active infection.

The field currently lacks the necessary data to measure the burden of coccidioidomycosis. Previously, an estimate of 150 000 total annual US asymptomatic and symptomatic *Coccidioides* infections was proposed; however, a robust calculation was not conducted.^17^ We endeavored to generate a systematic and rigorous estimate of national incident symptomatic coccidioidomycosis cases by developing a model to account for cases that are missed through national surveillance.

## Methods

### Data Source

We obtained reported coccidioidomycosis case counts from January 1 to December 31, 2019, through the National Notifiable Diseases Surveillance System (NNDSS), the most recent year of pre–COVID-19 data available at the time of analysis. For states where coccidioidomycosis is reportable (Figure, A, and eAppendix in Supplement 1), state health departments use passive surveillance to submit yearly case data to the NNDSS when a person meets laboratory and/or clinical criteria according to the Council of State and Territorial Epidemiologists case definition.^18^ Due to the COVID-19 pandemic, 2019 coccidioidomycosis NNDSS data for California were incomplete; therefore, California submitted case surveillance data directly to the Mycotic Diseases Branch of the Centers for Disease Control and Prevention (CDC). For states where coccidioidomycosis is not a reportable disease, cases were approximated using 2019 US Census population estimates and mean incidence rates from reporting states within the same level of endemicity (Figure, A) to calculate estimated cases that could be reported to public health entities if coccidioidomycosis was reportable. Endemicity levels were categorized as high (Arizona and California), low (Nevada, New Mexico, Texas, Utah, and Washington), or unknown (all other states and Washington, DC) based on public health surveillance data, skin test studies, outbreaks, and case reports (Figure, A).^19,20,21,22^ Geographic location of cases was based on patient residence and not necessarily location of exposure or location of diagnosis; travel history was not available. This activity was reviewed by the CDC and was conducted consistent with applicable federal law and CDC policy (for example, 45 CFR §46.102(l)(2),^23^ 21 CFR §56^24^; 42 USC §241(d); 5 USC §552a; 44 USC §3501 et seq). Owing to the use of deidentified data, informed consent was not required. We followed the Strengthening the Reporting of Observational Studies in Epidemiology (STROBE) reporting guideline for cross-sectional studies. Data were accrued from January 1, 2022, to July 1, 2024, and analyzed from October 1, 2022, to September 1, 2024.

**Figure.**
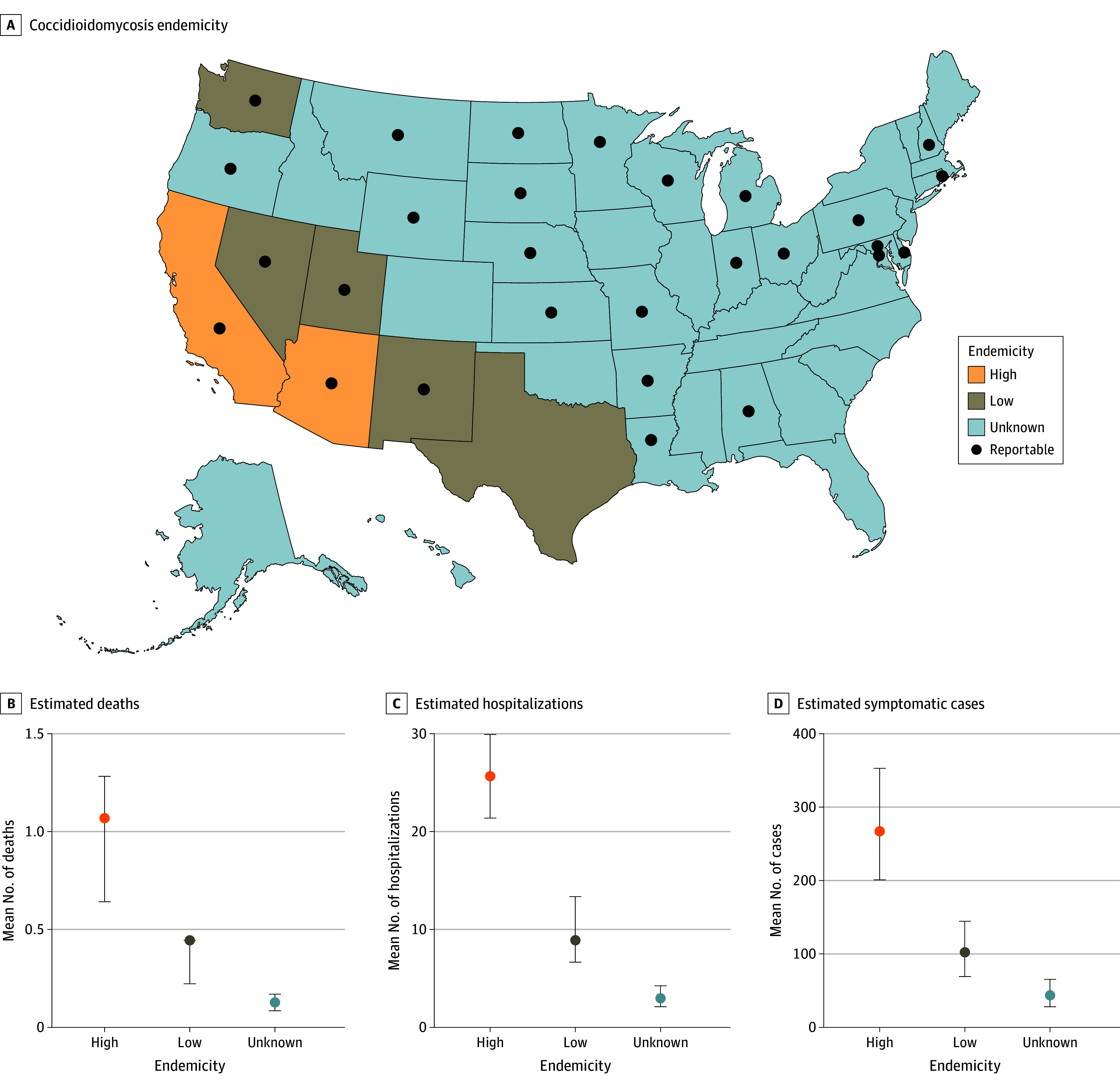
Coccidioidomycosis Deaths, Hospitalizations, and Symptomatic Cases by Endemicity A, Map of states by categorized coccidioidomycosis endemicity and reportable status as of 2019. B, Estimated coccidioidomycosis-associated deaths per 100 000 population. C, Estimated coccidioidomycosis-associated hospitalizations per 100 000 population. D, Estimated symptomatic coccidioidomycosis cases per 100 000 population. Dots represent mean estimates; bars, 95% credible intervals.

### Multipliers

Each multiplier used in the model either expanded or contracted the case counts to produce the final burden estimate. Values for the percentage of incident symptomatic cases among persons seeking health care were separated based on severity of disease (uncomplicated vs severe); values for the percentage of incident symptomatic cases reported to public health entities and the percentage of reported cases that are symptomatic were separated based on endemicity; and values for the percentage of incident symptomatic cases diagnosed accurately were separated based on severity of disease and endemicity. We explicitly accounted for the uncertainty of each multiplier using a distribution of plausible values and 100 000 simulations via Monte Carlo methods.

Most multiplier values were obtained from literature or previous enhanced surveillance projects (eAppendix in Supplement 1). To obtain a value for the percentage of incident symptomatic cases reported to public health entities in high-endemic states, the Arizona Department of Health Services matched 2019 case surveillance data submitted to its statewide surveillance system with serologic testing data reported by a large commercial laboratory in Arizona.

Three multiplier values were unavailable from published literature: (1) percentage of incident symptomatic case patients seeking health care, (2) percentage of incident symptomatic cases diagnosed accurately, and (3) percentage of incident symptomatic cases reported to public health surveillance. Epidemiologic data will unlikely be available in the immediate future, as measurement of these factors is inherently challenging and requires substantial resources. We therefore solicited values for these multipliers using expert opinion incorporating similar methods that were used in analyses for other diseases.^25,26^

We reached out to a convenience sample of 17 experts, with 12 clinicians (including A.S., A.C., I.S.S., L.M.-G., N.C.B., R.K., A.H., G.R.T., R.J., and J.N.G.) and 5 public health officials (G.C., R.H.S., B.O., H.N.O., and R.R.R.). Selected clinicians are leading coccidioidomycosis experts in the field and were identified based on vast experience diagnosing and managing coccidioidomycosis or other dimorphic fungi. Further details regarding multipliers, expert opinion process, and the modeling approach can be found in the eAppendix in Supplement 1.

### Statistical Analysis

#### Incident Symptomatic Cases Model

The incident symptomatic cases model scaled up from reported or approximated cases using a series of multipliers to generate an estimate of incident symptomatic cases based on the product of the covariates. The model extrapolated reported case counts to account for underreporting, underdiagnosis, and symptomatic case patients who did not seek health care:

ISC = (Cases × UR × PS) × {[PH × UD(Severe) × SC(Severe)] + 

[(1 – PH) × UD(Uncomplicated) × SC(Uncomplicated)]}

where ISC indicates incident symptomatic cases; Cases, reported or approximated case counts obtained from public health reporting systems; UR, underreporting, an expansive multiplier to account for the number of cases not reported to public health entities (ie, the reciprocal of the percentage of cases reported to public health entities); PS, percentage symptomatic, a contractive multiplier to represent the percentage of cases reported to public health entities that are symptomatic; PH, percentage hospitalized, a contractive multiplier to represent the percentage of cases in hospitalized patients; UD, underdiagnosis, an expansive multiplier to account for the number of cases that are misdiagnosed (ie, the reciprocal of the percentage of cases diagnosed accurately); and SC, seeking care, an expansive multiplier to account for the number of cases that do not seek health care (ie, the reciprocal of the percentage of cases in patients who seek health care). Severe cases were defined as patients who were hospitalized. We used a Poisson sampling distribution for reported cases and a Monte Carlo approach (100 000 iterations) to produce posterior distributions and generate the model output, represented by a mean point estimate of incident symptomatic cases and 95% credible intervals (CrIs). Estimates were rounded to the nearest thousand.

#### Hospitalizations Model

The coccidioidomycosis-associated hospitalizations model was similar to the incident symptomatic cases model but was limited to severe cases and the corresponding multipliers:

Hospitalizations = Cases × UR × PS × PH × UD(Severe) × SC(Severe).

Estimates were rounded to the nearest thousand.

#### Deaths Model

To estimate coccidioidomycosis-related deaths, we applied a literature-based proportion for in-hospital mortality to the estimated hospitalizations. We assumed that only severe disease would result in death, and therefore multipliers for severe disease were applied:

Deaths = Cases × UR × PS × PH × UD(Severe) × SC(Severe) × IHM

where IHM indicates in-hospital mortality, a contractive multiplier to represent in-hospital mortality rate. Estimates were rounded to the nearest hundred. Analyses were completed in RStudio, version 4.0.3 (R Program for Statistical Computing).

## Results

### Multipliers

Based on the values obtained through the expert opinion process, we estimated that 30% (IQR, 21%-32%) of people with symptomatic uncomplicated coccidioidomycosis and 99% (IQR, 98%-100%) of people with severe coccidioidomycosis sought health care. We similarly estimated the percentage of patients seeking care who were diagnosed accurately for uncomplicated disease (high endemicity, 38% [IQR, 24%-43%]; low endemicity, 20% [IQR, 10%-23%]; unknown endemicity, 8% [IQR, 3%-10%]) and severe disease (high endemicity, 80% [IQR, 58%-83%]; low endemicity, 45% [IQR, 28%-50%]; unknown endemicity, 25% [IQR, 9%-30%]). Through the expert review process, it was estimated that 32% (IQR, 13%-53%) of accurately diagnosed coccidioidomycosis cases were reported to public health entities in low-endemic states and 13% (IQR, 10%-25%) were reported in states of unknown endemicity. Based on the comparison of Arizona surveillance data and serologic laboratory testing data, we estimated that 80% of accurately diagnosed cases (based on positive laboratory results) were reported to public health entities in high-endemic states.

Details regarding all multiplier values, their sources, and uncertainty parameters can be found in the eTable in Supplement 1. These values served as model inputs to generate point estimates and 95% CrIs for incident symptomatic cases, hospitalizations, and deaths associated with coccidioidomycosis.

### Burden Estimates

We estimated that 273 000 (95% CrI, 206 000-360 000) incident symptomatic coccidioidomycosis cases occurred in 2019. The greatest estimated burden was attributed to high-endemic states (125 000; 95% CrI, 94 000-165 000), followed by states with unknown endemicity (103 000; 95% CrI, 66 000-155 000) and low-endemic states (46 000; 95% CrI, 31 000-65 000) (Table). Estimates translated to a national incidence of 83 (95% CrI, 63-110) cases per 100 000 population. We estimated a regional incidence of 267 (95% CrI, 201-353) cases per 100 000 population in high-endemic states, 102 (95% CrI, 69-144) cases per 100 000 population in low-endemic states, and 44 (95% CrI, 28-66) cases per 100 000 population in states of unknown endemicity (Figure, D).

**Table.** Estimated Total Annual Coccidioidomycosis Incident Symptomatic Cases, Hospitalizations, and Deaths in the US in 2019

Region	Estimated No. (95% CrI)		
	Incident symptomatic cases	Hospitalizations	Deaths
High-endemic states^a^	125 000 (94 000-165 000)	12 000 (10 000-14 000)	500 (300-600)
Low-endemic states^b^	46 000 (31 000-65 000)	4000 (3000-6000)	200 (100-200)
States of unknown endemicity^c^	103 000 (66 000-155 000)	7000 (5000-10 000)	300 (200-400)
All	273 000 (206 000-360 000)	23 000 (18 000-28 000)	900 (700-1100)

Abbreviation: CrI, credible interval.

^a^ Includes Arizona and California.

^b^ Includes New Mexico, Nevada, Texas, Utah, and Washington.

^c^ Includes all other states and Washington, DC.

An estimated 23 000 (95% CrI, 18 000-28 000) coccidioidomycosis-associated hospitalizations occurred in 2019. High-endemic states accounted for half of the total estimated hospitalizations (12 000; 95% CrI, 10 000-14 000), while 7000 (95% CrI, 5000-10 000) and 4000 (95% CrI, 3000-6000) hospitalizations were estimated in states with unknown endemicity and low-endemic states, respectively (Table). We estimated 7 (95% CrI, 5-9) hospitalizations per 100 000 population nationwide; regionally, we estimated 26 (95% CrI, 21-30) hospitalizations per 100 000 population in high-endemic states, 9 (95% CrI, 7-13) hospitalizations per 100 000 population in low-endemic states, and 3 (95% CrI, 2-4) hospitalizations per 100 000 population in states of unknown endemicity (Figure, C).

We estimated that 900 (95% CrI, 700-1100) coccidioidomycosis-related deaths occurred in 2019. Most estimated deaths occurred in high-endemic states (500; 95% CrI, 300-600), followed by states with unknown endemicity (300; 95% CrI, 200-400) and low-endemic states (200; 95% CrI, 100-200) (Table). We estimated 0.3 (95% CrI, 0.2-0.3) deaths per 100 000 population nationally with regional rates estimated at 1.1 (95% CrI, 0.6-1.3) deaths per 100 000 population in high-endemic states, 0.4 (95% CrI, 0.2-0.4) deaths per 100 000 population in low-endemic states, and 0.1 (95% CrI, 0.1-0.2) deaths per 100 000 population in states of unknown endemicity (Figure, B).

## Discussion

We estimated the annual burden of coccidioidomycosis in the US to be approximately 10 to 18 times higher than the 20 003 reported cases in 2019. The projected 206 000 to 360 000 incident symptomatic cases in 2019 translates to an incidence of approximately 58 to 117 per 100 000 population. As expected, the estimated burden was not evenly distributed geographically, with high-endemic states accounting for most cases. More than one-third of total cases were estimated in states not known to be endemic, primarily driven by national population totals. Among states where coccidioidomycosis is reportable, national surveillance captured 1 in 6 estimated cases from high-endemic states, 1 in 28 estimated cases from low-endemic states, and 1 in 123 estimated cases from states not known to be endemic.

Our burden estimate exceeds the previous and widely cited estimate of 150 000 symptomatic and asymptomatic cases per year.^17^ Presuming 40% of coccidioidomycosis infections are symptomatic, the estimated symptomatic case burden was 9 times higher than the burden reported by Galgiani et al,^17^ which is slightly below our estimated range. The reason for the higher estimated burden is likely multifaceted, but may be primarily driven by the more than 3-fold rise in reported cases since the previous estimate was published in 2005.^5^

Increased reporting is essential to enhance our understanding of coccidioidomycosis epidemiology and allow for more accurate characterization of trends, particularly in low-endemic states and states where coccidioidomycosis is not known to be endemic, considering that coccidioidomycosis is only reportable in 50% of all states. This dearth of case reporting contributes to the vast differences between estimated and reported case burden in states of low or unknown endemicity.^7^ More complete reporting and adding coccidioidomycosis as a reportable condition in the remaining states would strengthen the ability to monitor the spread of *Coccidioides* species, support estimations of geographic distribution, and better inform clinicians.

Overall, we approximate that nearly 60% of all estimated patients with coccidioidomycosis seek care (30% of patients with uncomplicated coccidioidomycosis and 99% of patients with severe disease), which provides valuable insight into the potential health care burden from a clinical standpoint. From a public health perspective, it is also essential to account for cases in patients who do not seek care, as the reasons for medical care avoidance are unclear. For example, while some patients with uncomplicated coccidioidomycosis may not seek medical care, others may have relatively inconsequential symptoms, and patients with both levels of severity are combined for this analysis. Notably, even mild cases could result in reactivation of latent infection, particularly for people who become immunosuppressed months or even years after the initial infection.^27^ Factors such as income, access to care, comfort with medical engagement, and insurance status may also influence health care–seeking behavior.^28,29,30^

Estimated hospitalizations were 2 to 3 times greater than the 9300 total coccidioidomycosis-associated inpatient stays documented by *International Statistical Classification of Diseases, Tenth Revision, Clinical Modification* (*ICD-10-CM*) codes in the Healthcare Cost and Utilization Project National Inpatient Sample (NIS).^31^ Although the NIS is designed to produce national estimates of inpatient stays in the US, it is subject to limitations of misdiagnosis and underrepresentation of coccidioidomycosis in *ICD-10-CM* codes, so the true number of coccidioidomycosis hospitalizations likely exceeds the total recorded in the NIS. Similarly, estimated deaths were 5 to 6 times higher than the 192 death records where coccidioidomycosis was listed as a contributing or underlying cause of death in the National Vital Statistics System Multiple Cause of Death Database.^32^ A previous study using capture-recapture methods found that death registry data underreported coccidioidomycosis-related deaths by 2- to 7-fold in Arizona.^33^

Improvements in early and accurate diagnosis are needed to enhance patient treatment and health outcomes and advance characterization of disease burden.^6^ Overall, rates of coccidioidomycosis testing are low for patients presenting with CAP, which can lead to low perceived prevalence despite reports that *Coccidioides* species cause approximately 15% to 30% of CAP in certain endemic regions.^12,13,34,35,36^ Testing rates are higher in areas where *Coccidioides* is known to reside in the soil, but it is important to retain clinical suspicion in regions outside the known endemic range to account for travel-associated cases or unexpected local acquisition.^12,13^ Increased awareness nationwide is needed among clinicians and the general public, as patients with knowledge of coccidioidomycosis may be more likely to be diagnosed earlier than those unfamiliar with the disease.^3^ Prompt diagnosis can accelerate proper disease management to mitigate the effect of coccidioidomycosis on activities of daily living and help to reduce the financial burden of unnecessary health care visits and ineffective antibacterial treatment.

### Limitations

Our work is subject to several limitations. The NNDSS data used as the primary model input do not capture all cases, since coccidioidomycosis is only reportable in a subset of states (Figure, A, and eAppendix in Supplement 1) and therefore required approximation based on assumed endemicity for states where the disease is not reportable. The estimates also relied on a series of multiplier values, several of which necessitated expert opinion, which may be subject to bias and do not necessarily represent the expert opinion of the entire field; the lack of data for validation also impedes our ability to assess the direction of bias and whether cases represent underestimation or overestimation. We minimized potential biases by asking experts to submit point estimates without conferring with other experts. Experts had varying backgrounds, which likely influenced opinion, and some of the range of expert values was wide. Additionally, multipliers obtained through literature were generally based on only a few studies. The geographic distribution of cases was based on residence, which does not necessarily align with potential exposure location (eg, we were unable to account for travel history). Lack of granular data also required endemicity-specific multipliers (percentage reported and percentage diagnosed accurately) to be applied uniformly at the state level, despite wide variations in endemicity across some states. Last, our estimates do not account for demographic, medical, or exposure-related risk factors or lifelong immunity. Despite these limitations, our estimates leverage the best sources of case and epidemiologic data currently available.

More robust multiplier values are needed to help refine future estimations, as multipliers substantially impact the final burden estimates. Although we recognize the limitations of evidence supplied through expert opinion, in the absence of superior data, this approach has proved useful in burden estimates and provides an efficient and low-resource means of obtaining meaningful data points.^25,26^

## Conclusions

In this cross-sectional study, we sought to provide a comprehensive and systematic estimate of coccidioidomycosis burden to raise public and clinician awareness and to inform clinical, public health, and policy decision-making. Our results highlight the need for increased awareness, accurate clinical diagnosis, and reporting to public health entities to improve patient outcomes and monitor trends over time, particularly in areas of low or unknown endemicity. Improved routine surveillance, enhanced surveillance projects, and targeted studies are essential to produce more reliable multipliers from primary data. We hope these estimates will be a first step in a long and sustained iterative effort to understand the burden of coccidioidomycosis in the US.

## Notes

Supplement 1. **eAppendix.** Multipliers and Modeling Approach **eTable.** Model Inputs and Parameters for Uncertainty

Supplement 2.
Data Sharing Statement
