# Error in Equation

**DOI:** 10.1001/jamanetworkopen.2025.25545

**Published:** 2025-07-14

In the Original Investigation titled “Estimated Burden of Coccidioidomycosis,” published on June 3, 2025, the equation on page 4 contained typographical errors in the use of parentheses and brackets. This article has been corrected online.^1^
