# Supplementary material for: Estimated Burden of Coccidioidomycosis
Source: JAMA Netw Open. 2025 Jun 3;8(6):e2513572. doi: 10.1001/jamanetworkopen.2025.13572 (PMC12134948; doi:10.1001/jamanetworkopen.2025.13572)
Supplement: Supplement 1. — eAppendix. Multipliers and Modeling Approach eTable. Model Inputs and Parameters for Uncertainty [file jamanetwopen-e2513572-s001.pdf]

Supplementary Online Content

Williams SL, Benedict K, Jackson BR, et al. Estimated burden of coccidioidomycosis in the US. *JAMA Netw Open.* 2025;8(6):e2513572. doi:10.1001/jamanetworkopen.2025.13572

eAppendix. Multipliers and Modeling Approach
eTable. Model Inputs and Parameters for Uncertainty

This supplementary material has been provided by the authors to give readers additional information about their work.

© 2025 Williams SL et al. *JAMA Network Open*.

eAppendix. Multipliers and Modeling Approach

Expert opinion multipliers

We sought out several multiplier values because they were not available through literature review: Percent of cases reported to public health (PR), percent of cases diagnosed accurately (PD), and percent of cases who sought care (PSC). Multipliers are essential components of the model because PR, PD, and PSC heavily influenced the final estimates. As a result, we carefully selected methods to obtain unbiased expert opinion of these multipliers using a multipronged approach.

We reached out to a convenience sample of 17 experts, which included 12 clinicians and 5 public health officials. Selected clinicians are leading coccidioidomycosis experts in the field and were identified based on vast experience diagnosing and managing patients with coccidioidomycosis in high-endemic regions. Clinicians in non-endemic regions demonstrated vast experience diagnosing and managing patients with other dimorphic fungi (e.g., histoplasmosis, blastomycosis). Public health officials were selected based on extensive experience conducting routine coccidioidomycosis surveillance. To ensure representation across various levels of endemicity, we selected five clinicians and two public health officials from high-endemic areas; two clinicians and two public health officials from low-endemic areas; and five clinicians and one public health official from in areas of unknown endemicity. Experts individually and blindly submitted point estimates for each multiplier without conferring with other experts and additionally indicated if they were uncertain about the values provided; any estimates where the expert was uncertain were excluded from the analysis. The median expert values served as the multipliers in the multiplicative model. For each multiplier, we used the beta–PERT (Program Evaluation and Review Technique) distribution to explicitly account for uncertainty.

© 2025 Williams SL et al. *JAMA Network Open*.

An initial group of 10 coccidioidomycosis experts submitted estimated values for PR, PD, and PSC. The expert group comprised five clinicians and five public health officials; seven experts worked in high-endemic areas (all five clinicians, two public health officials), two public health officials worked in low-endemic areas, and one public health official worked in an area of unknown endemicity. Regarding PR and PD, experts were asked to submit values for each endemicity level (high, low, and unknown); for PSC, experts submitted values based on severity. Experts were initially not asked to differentiate PD by disease severity.

Median values from the initial expert group were used to produce preliminary estimates of coccidioidomycosis burden. These preliminary multiplier values and burden estimates were presented at the 67th Annual Coccidioidomycosis Study Group (CSG) on March 31, 2023, in Tucson, Arizona, which convened an interdisciplinary group of coccidioidomycosis experts. CSG attendees were asked to participate in an interactive polling exercise conducted through Poll Everywhere to indicate whether they felt each value was too high, about right, or too low. More than half of the 73 respondents felt that the PD multiplier value was too low.

Following the CSG polling, additional input for the PD multiplier was requested from seven new clinicians (two who worked in low-endemic states and five who worked in states not known to be endemic), and were asked to provide distinct values based on endemicity level and disease severity (uncomplicated and hospitalized). We also asked the initial group of clinician experts to estimate separate PD values for uncomplicated versus hospitalized disease. The final multiplier values for PD included the median values that were obtained after the CSG.

Multiplier uncertainty estimates

© 2025 Williams SL et al. *JAMA Network Open*.

Wide uncertainty, both of documented literature values as well as expert opinion, is inherent in the construction of the model. Therefore, uncertainty was explicitly accounted for throughout the structure of these models. We used the beta–PERT (Program Evaluation and Review Technique) distribution as our basic descriptive distribution that the multipliers were assumed to follow. Beta-PERT allows intuitive input of user-defined parameters (minimum, modal value, and maximum, and a fourth parameter that controls the spread/variance of the distribution) to describe the distribution of a given variable, which is useful for modeling expert data. We specified four parameters: the most likely value (point estimate obtained from literature or median value obtained through expert opinion), the lowest and highest estimates (using the lower and upper values of the quantity of interest when available or the lower and upper bounds of the calculated 95% CI), and the shape or scale value (default values set at 4).

Model approach

To produce an estimate of coccidioidomycosis burden in the United States, we developed a multiplicative model that scaled up from cases reported to the National Notifiable Diseases Surveillance System (NNDSS). At the time of analysis, 27 states and the District of Columbia reported coccidioidomycosis cases to NNDSS: Alabama, Arizona, Arkansas, California, Delaware, Indiana, Kansas, Kentucky, Louisiana, Maryland, Michigan, Minnesota, Missouri, Montana, Nebraska, Nevada, New Hampshire, New Mexico, North Dakota, Ohio, Oregon, Rhode Island, South Dakota, Utah, Washington, Wisconsin, Wyoming. We applied a series of multipliers to account for underreporting, underdiagnosis, and patients who do not seek care. Each associated multiplier value represents a proportion of the input case data, and the reciprocal values are used as expansive factors in the model (e.g. underreporting (UR) and underdiagnosis (UD)). Values related to underreporting and underdiagnosis were distinct based on

© 2025 Williams SL et al. *JAMA Network Open*.

endemicity, and values related to underdiagnosis and care-seeking behavior were distinct based on disease severity.

Some multipliers were used as contractive factors to subset a certain proportion of cases. Such multipliers included percent uncomplicated and percent severe disease, and the appropriate underdiagnosis and care-seeking multipliers were subsequently applied. Additionally, the proportion of patients from states of low or unknown endemicity who first sought care in a high-endemic state were modeled using the PD multipliers for high-endemic states.

We summarized the outputs of our models using posterior distributions calculated by Monte Carlo simulation, with 100,000 replicates. For each iteration, a random sample was drawn from each model input distribution, then multiplied to produce the final estimate.

© 2025 Williams SL et al. *JAMA Network Open*.

eTable. Model Inputs and Parameters for Uncertainty

**Model Input**	**Data Source**	**Value/Parameters (low, model, high values)***
Percent seeking care – uncomplicated	30% of patients with uncomplicated coccidioidomycosis are assumed to seek care, based on expert opinion.	0.22, 0.30, 0.39
Percent seeking care – severe	99% of patients with severe coccidioidomycosis are assumed to seek care, based on expert opinion.	0.99, 0.99, 0.99
Percent reported to public health[§] – high endemicity	A match between 2019 Medical Electronic Disease Surveillance Intelligence System data in Arizona and serologic testing data reported by electronic laboratory report from a large commercial laboratory estimated that 80% of positive laboratory results are reported to public health in high-endemic states.	0.73, 0.80, 0.90
Percent reported to public health[§] – low endemicity	32% of coccidioidomycosis cases are assumed to be reported to public health in low-endemic states, based on expert opinion.	0.24, 0.32, 0.41
Percent reported to public health[§] – unknown endemicity	13% of coccidioidomycosis cases are assumed to be reported to public health in states of unknown endemicity, based on expert opinion.	0.09, 0.13, 0.18
Percent reported to public health symptomatic – high endemicity	Cooksey et al., 2024 (unpublished data) found that 86% of reported coccidioidomycosis cases in an enhanced surveillance study in California were symptomatic. Tsang et al., 2007 found that 95% of reported coccidioidomycosis cases in an enhanced surveillance study in Arizona were symptomatic.	0.87, 0.91, 0.94
Percent reported to public health symptomatic – low endemicity	Benedict et al., 2018 found that 86% of reported coccidioidomycosis cases in enhanced surveillance study were symptomatic in low-endemic states.	0.80, 0.86, 0.90
Percent reported to public health symptomatic – unknown endemicity	Benedict et al., 2018 found that 94% of reported coccidioidomycosis cases in enhanced surveillance study were symptomatic in states of unknown endemicity.	0.91, 0.94, 0.96
Percent diagnosed accurately[†] – uncomplicated, high endemicity	38% of patients with uncomplicated coccidioidomycosis are assumed to be diagnosed accurately in high-endemic states, based on expert opinion.	0.29, 0.38, 0.48
Percent diagnosed accurately[†] – uncomplicated, low endemicity	20% of patients with uncomplicated coccidioidomycosis are assumed to be diagnosed accurately in low-endemic states, based on expert opinion.	0.14, 0.20, 0.27
Percent diagnosed accurately[†] – uncomplicated, unknown endemicity	8% of patients with uncomplicated coccidioidomycosis are assumed to be diagnosed accurately in states of unknown endemicity, based on expert opinion.	0.05, 0.08, 0.12
Percent diagnosed accurately[†] – severe, high endemicity	80% of patients with severe coccidioidomycosis who seek care are assumed to be diagnosed accurately in high-endemic states, based on expert opinion.	0.73, 0.80, 0.86
Percent diagnosed accurately[†] – severe, low endemicity	45% of patients with severe coccidioidomycosis who seek care are assumed to be diagnosed accurately in low-endemic states, based on expert opinion.	0.35, 0.45, 0.55
Percent diagnosed accurately[†] – severe, unknown endemicity	25% of patients with severe coccidioidomycosis who seek care are assumed to be diagnosed accurately in states of unknown endemicity, based on expert opinion.	0.15, 0.25, 0.33
Percent uncomplicated disease	Tsang et al., 2007 found that 59% of coccidioidomycosis cases in an enhanced surveillance study from a high-endemic state were not hospitalized. Benedict et	0.48, 0.58, 0.67

© 2025 Williams SL et al. *JAMA Network Open*.

	al., 2018 found that 56% of coccidioidomycosis cases in an enhanced surveillance study from states of low or unknown endemicity were not hospitalized. A mid-point estimate from these two studies was used as the model value.	
Percent severe disease	Tsang et al., 2007 found that 41% of coccidioidomycosis cases in an enhanced surveillance study from a high-endemic state were not hospitalized. Benedict et al., 2018 found that 44% of coccidioidomycosis cases in an enhanced surveillance study from states of low or unknown endemicity were not hospitalized. A mid-point estimate from these two studies was used as the model value.	0.33, 0.42, 0.52
Percent patients from low-endemic states who first sought care in high-endemic states	Benedict et al., 2018 (unpublished data) found that 4% of coccidioidomycosis patients from low-endemic states in an enhanced surveillance study first sought care in a high-endemic state.	0.03, 0.04, 0.06
Percent patients from states of unknown endemicity who first sought care in high-endemic states	Benedict et al., 2018 (unpublished data) found that 44% of coccidioidomycosis patients from states of unknown endemicity in an enhanced surveillance study first sought care in a high-endemic state.	0.34, 0.44, 0.54
In-hospital mortality rate	Data from the 2019 Healthcare Cost and Utilization Project National Inpatient Sample showed that 4.1% of patients with coccidioidomycosis-associated hospitalizations died in-hospital. The database showed that in-hospital mortality rates ranged from 2.7% to 4.4% between 2010–2020; these were used as the low and high values.	0.03[‡], 0.04, 0.04[‡]

*High/low values were based on a 50% relative increase/decrease from the modal value on an odds scale
§For reporting multipliers, it was assumed that patients had sought care and cases had been diagnosed accurately
†For diagnosis multipliers, it was assumed that patients had sought care
‡Low and high values determined based on minimum, average, and maximum findings from Luo et al., 2017

© 2025 Williams SL et al. *JAMA Network Open*.
