# Supplementary material for: Estimated Burden of Coccidioidomycosis
Source: JAMA Netw Open. 2025 Jun 3;8(6):e2513572. doi: 10.1001/jamanetworkopen.2025.13572 (PMC12134948; doi:10.1001/jamanetworkopen.2025.13572)
Supplement: Supplement 2. — Data Sharing Statement [file jamanetwopen-e2513572-s002.pdf]

Data Sharing Statement

Williams. Estimated Burden of Coccidioidomycosis in the US. *JAMA Netw Open*. Published June 03, 2025. doi:10.1001/jamanetworkopen.2025.13572

Data
Data available: Yes
Data types: Data (not involving human participants)
How to access data: https://wonder.cdc.gov/nndss/static/2019/annual/2019-table2e.html
When available: beginning date: 01-01-2021

Supporting Documents
Document types: None

Additional Information
Who can access the data: Anyone. It's already publicly available.
Types of analyses: For any purpose.
Mechanisms of data availability: It is publicly accessible data.
